# Association of the triglyceride glucose-body mass index with the extent of coronary artery disease in patients with acute coronary syndromes

**DOI:** 10.1186/s12933-024-02124-2

**Published:** 2024-01-13

**Authors:** Xueyuan Yang, Kui Li, Jiaojiao Wen, Changlong Yang, Yunhang Li, Guanxue Xu, Yi Ma

**Affiliations:** Department of Cardiovascular Medicine, Affiliated Hospital of Zunyi Medical University, No. 149 Dalian Road, Zunyi, Guizhou 563099 China

**Keywords:** Acute coronary syndrome, Triglyceride glucose-body mass index, Cardiovascular disease, Insulin resistance, SYNTAX score

## Abstract

**Background:**

Studies have shown that insulin resistance is strongly associated with the development of cardiovascular disease, and the triglyceride glucose-body mass index (TyG-BMI index) is considered to be a reliable surrogate marker of insulin resistance. There are limited studies on the relationship between TyG-BMI index and the extent of coronary artery disease in patients with acute coronary syndrome (ACS). This study aimed to investigate the relationship between TyG-BMI index and the extent of coronary artery disease in patients with ACS.

**Methods:**

Overall, 2,317 patients with ACS who underwent percutaneous coronary intervention at the Affiliated Hospital of Zunyi Medical University were included in this study. The TyG-BMI index was grouped according to the tertile method. The extent of coronary artery disease in patients with ACS was quantitatively assessed using the SYNTAX score, which was categorised as low (≤ 22), intermediate (23–32), and high risk (≥ 33).

**Results:**

In the overall population, multivariate logistic regression analyses showed that TyG-BMI index was associated with mid/high SYNTAX score in patients with ACS (odds ratio [OR] = 1.0041; 95% confidence interval [CI] = 1.0000–1.0079; *p* = 0.0310). Subgroup analyses showed that TyG-BMI index was an independent risk factor for mid/high SYNTAX score in female ACS patients after adjusting for multiple confounders (OR = 1.0100; 95% CI = 1.0000–1.0200; *p* = 0.0050), and that the risk of mid/high SYNTAX score was 2.49 times higher in the T3 group (OR = 2.4900; 95% CI = 1.2200–5.0600; *p* = 0.0120). Restricted cubic spline analysis showed a linear correlation between TyG-BMI index and complex coronary artery disease (SYNTAX score > 22) in women with ACS. In female ACS patients, inclusion of the TyG-BMI index did not improve the predictive power of the underlying risk model (net reclassification improvement: 0.0867 [-0.0256–0.1989], *p* = 0.1301; integrated discrimination improvement: 0.0183 [0.0038–0.0329], *p* = 0.0135).

**Conclusions:**

TyG-BMI index is linearly associated with the degree of complex coronary artery disease in female ACS patients. However, the inclusion of the TyG-BMI index did not improve the predictive power of the underlying risk model for female ACS patients.

**Supplementary Information:**

The online version contains supplementary material available at 10.1186/s12933-024-02124-2.

## Background

Cardiovascular disease (CVD) is the leading cause of death worldwide, being especially notable in low- and middle-income areas, with nearly half of all patients with CVD dying from ischaemic heart disease [1]. Acute coronary syndromes (ACS), including unstable angina, acute non-ST-segment elevation myocardial infarction (NSTEMI), and acute ST-segment elevation myocardial infarction (STEMI), are a significant cause of death, with one person having a myocardial infarction (MI) approximately every 40 s in the United States [2, 3]. Recent European Society of Cardiology guidelines state that in patients with STEMI, urgent coronary artery bypass grafting (CABG) should be considered if the area of myocardial injury is large. Additionally, these guidelines state that there is no clear benefit of early versus delayed coronary angiography in patients with NSTEMI, and that appropriate ischaemia testing should be performed if there are no extremely high- or high-risk features and if there is a low index of suspicion for NSTEMI, before proceeding to further invasive methods [4]. However, there is a lack of non-invasive serological indicators for assessing the degree of coronary artery disease (CAD) in patients with ACS. Therefore, a search for more specific serological indicators for predicting the degree of CAD in patients with ACS can provide a reference for the assessment of their condition and is of great clinical significance for the development of therapeutic protocols.

Studies have shown that insulin resistance (IR) is an independent risk factor for vascular sclerosis and that it is associated with vascular sclerosis in all age groups [5]. The homeostatic model assessment (HOMA) has been shown to be a reliable method for describing IR [6]. Moreover, the triglyceride glucose index (TyG index) is a simple way to measure IR [7]. A recent study in a Brazilian population showed that the TyG index showed better ability to identify patients with IR than the HOMA score [8]. Another study in a Chinese population showed that the TyG index was a reliable indicator of IR and superior to the homeostatic model assessment of IR (HOMA-IR) in Chinese patients with type 2 diabetes with lower body mass index (BMI) [9]. The TyG index has been shown to correlate with atherosclerotic CVD (ASCVD) and the degree of coronary vascular disease [10–17].

Obesity, as defined by the BMI, plays an important role in the development of IR [18]. As BMI increases, so does the risk of coronary atherosclerotic heart disease [19]. The TyG-BMI index, obtained by correcting the TyG index for BMI, presents new insights into the relationship between poor prognosis and CVD. A study based on a Korean population showed that the TyG-BMI index predicted IR and significantly outperformed the TyG index alone [20]. It has also been shown that TyG-BMI index is significantly associated with an increased risk of ASCVD [21]. Furthermore, in a middle-aged population without CVD, the TyG-BMI index was significantly associated with progression of coronary artery calcification [22]. A study showed a linear relationship between the TyG-BMI index and the occurrence of ischaemic stroke [23], while another study showed that the same index was associated with the severity of CAD [24]. However, few studies have focused on the relationship between the TyG-BMI index and extent of CAD in patients with ACS. Therefore, the present study aimed to investigate the relationship between the TyG-BMI index and the extent of CAD in patients with ACS and assess whether it can be used as a reliable predictor of the extent of CAD in this patient population.

## Methods

### Study design and population

**Fig. 1:**
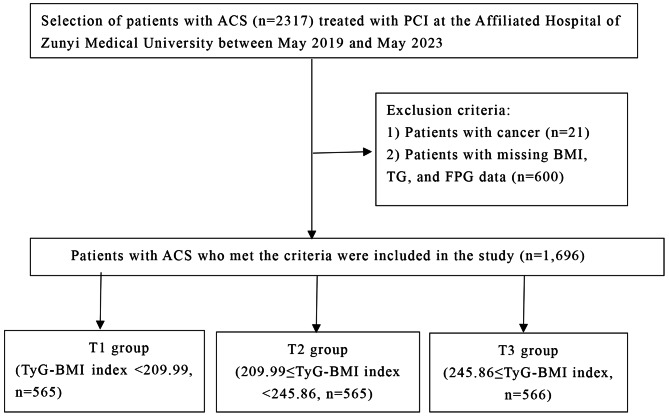
Study flowchart

This study was a cross-sectional study conducted at the Affiliated Hospital of Zunyi Medical University. The study complied with the Declaration of Helsinki and was authorised by the Ethics Committee of the Affiliated Hospital of Zunyi Medical University, which waived the requirement for informed consent due to its retrospective nature. Patients with ACS treated with percutaneous coronary intervention (PCI) at the Affiliated Hospital of Zunyi Medical University from 1 May 2019 to 1 May 2023 were included in this study. Patients meeting the following criteria were excluded: (1) history of malignancy; (2) missing BMI, triglycerides (TG), and fasting blood glucose (FBG) data (Fig. 1). Ultimately, a total of 1,696 patients with ACS treated with PCI were included in the final analysis. Patients were divided into three groups according to TyG-BMI index tertiles: group T1 (TyG-BMI index < 209.99), group T2 (209.99 ≤ TyG-BMI index < 245.86), and group T3 (245.86 ≤ TyG-BMI index). The extent of CAD in patients with ACS was quantitatively assessed using the SYNTAX score, according to which patients with CAD were categorised as being at low (≤ 22), intermediate (23–32), and high risk (≥ 33) [25]. Higher SYNTAX scores reflect more severe and complex disease. The primary endpoint was complex CAD (moderate/high SYNTAX score).

### Data measurement and definitions

Baseline demographic and clinical data of all patients were retrospectively collected from the medical records of the Affiliated Hospital of Zunyi Medical University. Demographic data included patient age; sex; BMI; smoking status; co-morbidities [hypertension, dyslipidaemia, diabetes mellitus (DM), and previous stroke]; family history; and history of MI, PCI, and CABG. Clinical data included patient systolic blood pressure (SBP) and diastolic blood pressure (DBP) on admission; primary diagnosis at admission (STEMI, NSTEMI, unstable angina pectoris [UA]), imaging data; laboratory tests and findings (left ventricular ejection fraction [LVEF], TG, total cholesterol [TC], and high-density lipoprotein cholesterol [HDL-C], low-density lipoprotein cholesterol [LDL-C], FBG, glycosylated haemoglobin [HbA1c], haemoglobin [Hb], creatinine [Cr], uric acid, and high-sensitivity C-reactive protein [hs-CRP]); with blood samples collected from all subjects in a fasting state. FBG and TG were determined using an AU5800 system (Beckman Coulter, Brea, CA). BMI was calculated as weight (Kg) divided by the square of height (m^2^). Estimated glomerular filtration rate (eGFR) was calculated according to the Modification of Diet in Renal Disease formula [26] for males as eGFR = 186 × Cr^− 1.154^ × age^− 0.203^ and females as eGFR = 186 × Cr^− 1.154^ × age^− 0.203^ × 0.742. The TyG-BMI index was defined as ln[TG (mg/dL) × FBG (mg/dL) /2] × BMI [27]. The presence of DM was defined as a history of type 2 diabetes or HbA1c ≥ 6.5% [28]. Elderly patients were defined as those aged ≥ 60 years [29]. SYNTAX scores were calculated from preoperative angiograms using a web-based online calculation tool (http://syntaxscore.com/).

### Statistical analysis

Statistical analyses were performed using R version 4.2.3 (R Foundation for Statistical Computing, Vienna, Austria). The mean ± standard deviation is used to describe continuous variables with a normal distribution, and the median (interquartile range) to describe continuous variables that are not normally distributed. Categorical variables are described as numbers (percentages). Comparisons between three groups of continuous variables that conformed to a normal distribution with chi-square variance were analysed using analysis of variance, and comparisons between three groups of continuous variables that did not conform to a normal distribution or chi-square variance were analysed using the Kruskal–Wallis rank-sum test. Comparisons of categorical values between the three groups were made using chi-square analysis. Logistic regression was used to analyse the correlation between TyG-BMI index and the severity of coronary artery lesions (SYNTAX score ≤ 22 or SYNTAX score > 22), and the odds ratio (OR) and 95% confidence interval (CI) were calculated. In the current study, model 1 was not adjusted. Model 2 was a multivariate model. Before constructing the multivariate model, we examined the collinearity between the TyG-BMI index and other covariates by calculating the generalised variance inflation factor (GVIF). The covariates were considered significant for collinearity if the GVIF^(1/2Df) was equal to or greater than 2 (Df, degrees of freedom). least absolute shrinkage and selection operator regression was then used to filter covariates from the pool of variables. The following covariates were adjusted for in the multivariate logistic regression analyses for the overall population: sex, age, smoking, Previous MI, hypertension, previous stroke, SBP, DBP, HDL-C, eGFR, creatinine, uric acid, hs-CRP, HbA1c, LVEF, insulin, and oral hypoglycemic drugs. Restricted cubic spline analyses were used to explore the relationship between TyG-BMI index and moderate/high SYNTAX scores. Subgroups were analysed according to the DM status, age, and sex, and univariate and multivariate logistic regression analyses were performed. Diagnostic value analyses were performed using receiver operating characteristic (ROC) curves, and the area under the curve (AUC), measured by the C-statistic, was calculated to quantify the predictive power of the logistic model for intermediate-/high-risk SYNTAX scores. AUC-based comparisons between models were assessed using the DeLong test. In addition, the Net Reclassification Index (NRI) and the Integrated Discriminant Improvement Index (IDI) were calculated to further assess the additional predictive value of the TyG-BMI index over and above the identified risk factors for moderate/high SYNTAX scores. *P* < 0.05 was considered statistically significant and all analyses were two-tailed.

## Results

### Baseline characteristics

A total of 1,696 patients with ACS treated with PCI were included in this study. The mean age of the patients was 62 (54,70) years; there were 1,207 (71.2%) male patients and 986 (58.1%) patients with DM. The patient age, sex, BMI, SBP, DBP, smoking status, hypertension grade, glycaemia, Hb, HbA1c, HDL-C, LDL-C, TC, TG, uric acid, insulin use, and oral hypoglycaemic drug use were differed between the three groups (all *P* < 0.05, Table 1). Patients in the T3 group had higher glucose, TC, TG, HbA1c, and BMI levels and more frequent history of insulin and oral hypoglycaemic drug use, history of DM, and hypertension than those in the other two groups.

**Table 1:** Baseline characteristics according to tertiles of the TyG-BMI index

	Total (*N* = 1,696)	T1 (*N* = 565)	T2 (*N* = 565)	T3 (*N* = 566)	*p*
Glycaemia(mmol/L)	7.69 (5.84,11.48)	6.30 (5.18,8.52)	7.57 (5.98,11.01)	9.99 (7.24,14.02)	< 0.001
Age (years)	62 (54,70)	65 (56,73)	63 (54,70)	57 (51,68)	< 0.001
BMI (kg/m^2^)	23.88 (21.97,26.45)	20.96 (19.72,22.49)	23.88 (22.86,25.35)	27.55 (25.64,29.55)	< 0.001
Creatinine(μmol/L)	78 (65,94)	76 (65,94)	78 (65,94)	79 (67,95)	0.083
hs-CRP(mg/L)	3.71 (1.21,14.30)	3.72 (1.27,15.74)	3.60 (1.13,12.80)	3.82 (1.36,14.30)	0.495
DBP (mmHg)	76 (68,85)	74 (67,82)	77 (68,86)	76 (68,85)	0.002
LVEF (%)	56 (46,61)	55 (45,60)	56 (46,60)	56 (49,61)	0.341
eGFR (mL/min/1.73 m^2^)	87.82 (70.43,105.09)	88.65 (69.51,105.09)	86.98 (69.71,103.97)	87.82 (70.97,106.53)	0.752
Hb (g/L)	139 (125,151)	134 (121,145)	139 (125,151)	145 (132,155)	< 0.001
HbA1c (%)	6.50 (5.80,8.00)	6.00 (5.60,7.00)	6.50 (5.70,8.00)	7.30 (6.10,8.98)	< 0.001
HDL-C(mmol/L)	1.09 (0.93,1.27)	1.14 (0.98,1.33)	1.10 (0.95,1.27)	1.05 (0.89,1.22)	< 0.001
LDL-C(mmol/L)	2.96 (2.35,3.60)	2.79 (2.22,3.48)	2.99 (2.36,3.55)	3.09 (2.49,3.76)	< 0.001
SBP (mmHg)	123 (112,136)	122 (110,135)	125 (112,136)	124 (111,138)	0.048
TC (mmol/L)	4.87 (3.99,5.76)	4.65 (3.80,5.53)	4.88 (4.01,5.67)	5.18 (4.12,6.06)	< 0.001
TG (mmol/L)	1.90 (1.26,2.91)	1.33 (0.98,1.94)	1.93 (1.36,2.75)	2.71 (1.82,4.22)	< 0.001
Uric acid(μmol/L)	354 (293,430)	342 (279,416)	357 (295,429)	366 (304,443)	0.001
Male	1,207 (71.2)	368 (65.1)	403 (71.3)	436 (77.0)	< 0.001
Smoking					< 0.001
Current	718 (42.3)	208 (36.8)	228 (40.4)	282 (49.8)	
Former	210 (12.4)	73 (12.9)	73 (12.9)	64 (11.3)	
Never	768 (45.3)	284 (50.3)	264 (46.7)	220 (38.9)	
Previous MI	556 (32.8)	185 (32.7)	178 (31.5)	193 (34.1)	0.649
Previous CABG	14 (0.8)	4 (0.7)	5 (0.9)	5 (0.9)	0.931
Previous PCI	197 (11.6)	67 (11.9)	62 (11.0)	68 (12.0)	0.841
Hypertension grade					0.002
1	101 (6.0)	32 (5.7)	32 (5.7)	37 (6.5)	
2	297 (17.5)	95 (16.8)	117 (20.7)	85 (15.0)	
3	643 (38.6)	186 (32.9)	215 (38.1)	242 (42.8)	
Dyslipidaemia	756 (44.6)	196 (34.7)	250 (44.2)	310 (54.8)	< 0.001
Previous stroke	211 (12.4)	59 (10.4)	84 (14.9)	68 (12.0)	0.074
Family history	8 (0.5)	3 (0.5)	4 (0.7)	1 (0.2)	0.414
Insulin	428 (25.2)	90 (15.9)	140 (24.8)	198 (35.0)	< 0.001
Oral hypoglycaemic drugs	758 (44.7)	179 (31.7)	252 (44.6)	327 (57.8)	< 0.001
DM	986 (58.1)	244 (43.2)	325 (57.5)	417 (73.7)	< 0.001
Diagnosis on admission					0.386
STEMI	426 (25.1)	132 (23.4)	153 (27.1)	141 (24.9)	
NSTEMI	432 (25.5)	155 (27.4)	129 (22.8)	148 (26.1)	
UA	838 (49.4)	278 (49.2)	283 (50.1)	277 (48.9)	
SYNTAX score	14 (8,19)	14 (8,19)	14 (8,19)	14 (8,19.5)	0.918

Data are presented as means ± SDs, medians (interquartile ranges), or n (%)

BMI, body mass index

### Clinical outcomes

In the overall population, multivariate logistic regression analyses showed that TyG-BMI index was associated with mid/high SYNTAX score in patients with ACS (OR = 1.0041; 95% CI = 1.0000–1.0079; *P* = 0.0310), and that in the T3 group (OR = 1.4700; 95% CI = 1.0100–2.1400; *P* = 0.0440) the risk of mid/high SYNTAX score was 1.47 times higher than that in the T1 group Table 2.

**Table 2:** Associations between the TyG-BMI index and moderate/high SYNTAX score

	Events/N	Model 1				Model 2				
		OR	95% CI	*p*		OR	95% CI		*p*	
TyG-BMI index	220/1,696	1.0027	0.9994–1.0061	0.1090		1.0041	1.0000-1.0079		0.0310	
T1	67/565	Reference				Reference				
T2	67/565	1.0000	0.6972–1.4343	1.0000		1.0100	0.6900–1.4700		0.9550	
T3	86/566	1.3300	0.9400–1.8800	0.1020		1.4700	1.0100–2.1400		0.0440	
*p* for trend				0.0954					0.0418	

### Subgroup analyses

Subgroup analyses of the correlation between TyG-BMI index and complex CAD in different populations according to DM status, sex, and age were conducted. Table 3 shows the relationship between TyG-BMI index and mid/high SYNTAX score in patients with different DM status. There was no significant interaction between DM subgroups and the effect of TyG-BMI index on mid/high SYNTAX score (interaction *P*-value = 0.9361). Furthermore, there was no statistically significant association between TyG-BMI index and mid/high SYNTAX scores in ACS patients in univariate logistic regression analyses and multivariate logistic regression analyses in either diabetic or non-diabetic populations.

**Table 3:** Associations between the TyG-BMI index and moderate/high SYNTAX score according to the diabetes status

	Events/N	Model 1				Model 2			
		OR	95% CI	*p*		OR	95% CI	*p*	*P* for interaction
Glucose metabolism state									0.9361
DM	119/986	1.0026	0.9981–1.0073	0.2590		1.0048	0.9998–1.0099	0.0610	
T1	25/244	Reference				Reference			
T2	36/325	1.0900	0.6400–1.8700	0.7510		1.2000	0.6800–2.1300	0.5260	
T3	58/417	1.4200	0.8600–2.3300	0.1720		1.7600	1.0200–3.0500	0.0420	
Non-DM	101/710	1.0049	0.9996–1.0103	0.0710		1.0044	0.9985–1.0104	0.1450	
T1	42/321	Reference				Reference			
T2	31/240	0.9900	0.6000–1.6200	0.9530		0.8300	0.4900–1.4000	0.4780	
T3	28/149	1.5400	0.9100–2.5900	0.1080		1.4000	0.8000–2.4600	0.2360	

Table 4 shows the relationship between TyG-BMI index and mid/high SYNTAX score in elderly/non-elderly patients. There was no significant interaction between age subgroups and the effect of TyG-BMI index on mid/high SYNTAX score (interaction *P*-value = 0.7923). Additionally, there was no statistically significant association between TyG-BMI index and mid/high SYNTAX scores in patients with ACS in univariate logistic regression analyses and multivariate logistic regression analyses in either the elderly or non-elderly population.

**Table 4:** Associations between the TyG-BMI index and moderate/high SYNTAX score according to age

	Events/N	Model 1				Model 2			
		OR	95% CI	*p*		OR	95% CI	*p*	*P* for interaction
Age-years									0.7923
Age ≥ 60	115/931	1.0032	0.9984–1.0081	0.1910		1.0052	0.9999–1.0104	0.0550	
T1	42/364	Reference				Reference			
T2	39/319	1.0700	0.6700–1.7000	0.7820		1.1400	0.7000–1.8500	0.5900	
T3	34/248	1.2200	0.7500–1.9800	0.4240		1.3600	0.8100–2.2900	0.2510	
Age < 60	105/765	1.0019	0.9970–1.0068	0.4470		1.0033	0.9978–1.0088	0.2420	
T1	25/201	Reference				Reference			
T2	28/246	0.9000	0.5100–1.6100	0.7310		0.8800	0.4800–1.6100	0.6690	
T3	52/318	1.3800	0.8200–2.3000	0.2230		1.6000	0.9100–2.8200	0.1050	

**Fig. 2:**
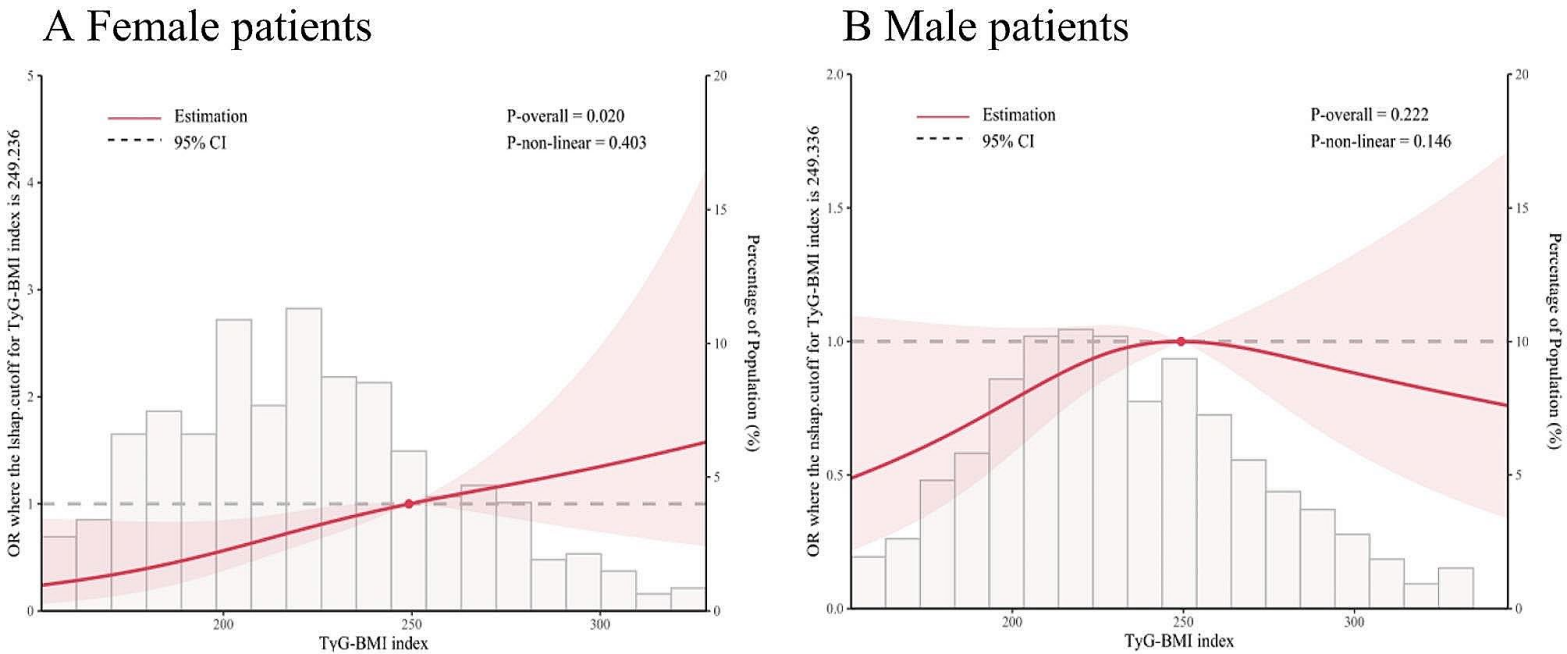
Restricted cubic spline curves for moderate/high SYNTAX score by TyG-BMI index after covariate adjustment. **A**, female patients; **B**, male patients. CI, confidence interval

Table 5 shows the relationship between TyG-BMI index and mid/high SYNTAX scores of patients of different genders. There was no significant interaction between gender subgroups and the effect of TyG-BMI index on mid/high SYNTAX score (interaction *p*-value = 0.0717). We found that TyG-BMI index was an independent risk factor for mid/high SYNTAX score in female ACS patients after adjusting for multiple confounders (OR = 1.0100; 95% CI = 1.0000–1.0200; *P* = 0.0050), and that the risk of mid/high SYNTAX score was 2.49 times higher in the T3 group (OR = 2.4900; 95% CI = 1.2200–5.0600; *P* = 0.0120) than that in the T1 group. For this purpose, we developed a restricted cubic spline model to define the nonlinear relationship between TyG-BMI index and complex coronary artery disease (mid/high SYNTAX score) in female ACS patients, and the model showed a linear association (nonlinear *p* = 0.403) (Fig. 2A). In the male subgroup, there was no significant correlation between TyG-BMI index and complex coronary artery disease, although the restricted cubic spline model showed a linear relationship (nonlinear *p* = 0.146) (Fig. 2B).

**Table 5:** Associations between the TyG-BMI index and moderate/high SYNTAX score according to sex

	Events/N	Model 1				Model 2			
		OR	95% CI	*p*		OR	95% CI	*p*	*P* for interaction
Sex									0.0717
Male	159/1,207	1.0007	0.9966–1.0047	0.7420		1.0023	0.9977–1.0069	0.3280	
T1	47/368	Reference				Reference			
T2	51/403	0.9900	0.6500–1.5100	0.9610		1.0300	0.6600–1.6100	0.8860	
T3	61/436	1.1100	0.7400–1.6700	0.6140		1.2600	0.8000–1.9700	0.3200	
Female	61/489	1.0076	1.0014–1.0138	0.0160		1.0100	1.0000-1.0200	0.0050	
T1	20/197	Reference				Reference			
T2	16/162	0.9700	0.4900–1.9400	0.9310		0.9300	0.4500–1.9300	0.8370	
T3	25/130	2.1100	1.1200–3.9800	0.0220		2.4900	1.2200–5.0600	0.0120	

CI, confidence interval; OR, odds ratio

### Incremental effect of TyG-BMI index in predicting moderate/high SYNTAX scores in female patients with ACS

**Fig. 3:**
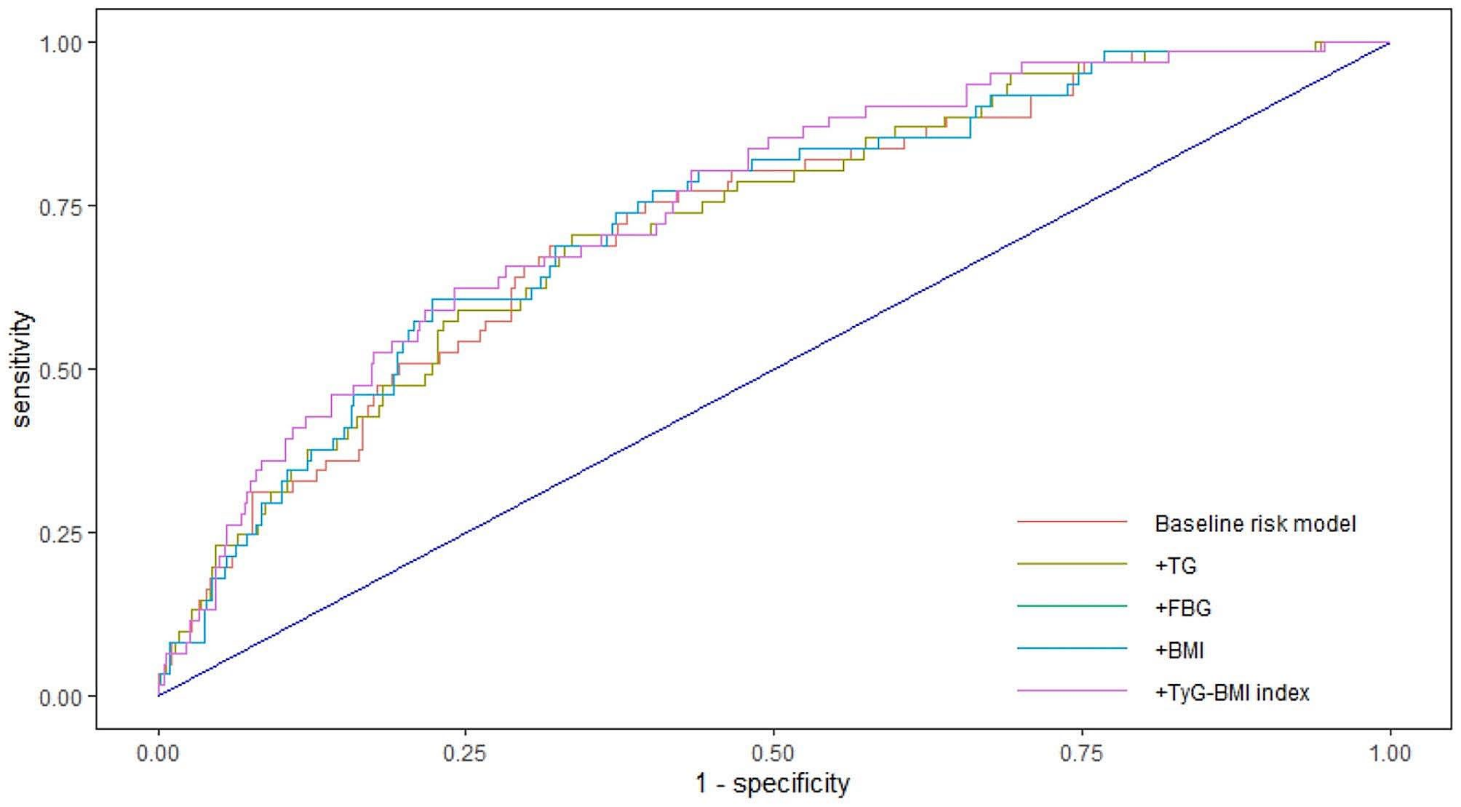
Receiver operating characteristic curves assessing the predictive ability of TG, FBG, BMI, and TyG-BMI index for a moderate/high SYNTAX score

In an analysis of female ACS patients treated with PCI, ROC curves were constructed to assess the predictive ability of the baseline risk model and the baseline risk model plus TG, FPG, BMI, and TyG-BMI index for the mid/high SYNTAX score, respectively (Fig. 3). There was no significant difference between the baseline risk model [AUC: 0.7211 (0.6547–0.7875)] and the TyG-BMI index model [AUC: 0.7488 (0.6858–0.8118)] (*P* = 0.0974). Table 6 lists the C-statistic, NRI, and IDI. No improvement was observed in the predictive power of the baseline risk model by incorporating the TyG-BMI index in female ACS patients treated with PCI (net reclassification improvement [NRI]: 0.0867 [-0.0256–0.1989], *P* = 0.1301; integrated discrimination improvement [IDI]: 0.0183 [0.0038–0.0329], *P* = 0.0135).

**Table 6:** Improvement in discrimination and risk reclassification for moderate/high SYNTAX score after adding TyG-BMI index in female patients

Model	C-statistic (95% Cl)	*P* value	NRI (95% Cl)	*P* value	IDI (95% Cl)	*P* value
Baseline risk model	0.7211(0.6547–0.7875)	Ref	Ref		Ref	
+TG	0.7231(0.6572–0.7889)	0.7758	0.0468(-0.0106-0.1043)	0.1101	0.0024(-0.0021-0.0069)	0.2931
+FBG	0.7303(0.6647–0.7959)	0.3212	0.0656(-0.0060-0.1373)	0.0727	0.0033(-0.0045-0.0111)	0.4019
+BMI	0.7482(0.6861–0.8104)	0.1116	0.0820(-0.0204-0.1844)	0.1165	0.0208(0.0051–0.0366)	0.0095
+TyG-BMI index	0.7488(0.6858–0.8118)	0.0974	0.0867(-0.0256-0.1989)	0.1301	0.0183(0.0038–0.0329)	0.0135

The baseline risk model included age, smoking, previous myocardial infarction, hypertension, previous stroke, SBP, DBP, HDL-C, eGFR, creatinine, uric acid, hs-CRP, HbA1c, LVEF, insulin, and oral hypoglycemic drugs.

## Discussion

Considering the lack of appropriate non-invasive serological indices for the clinical assessment of the extent of CAD in patients with ACS, we designed the present study to investigate the relationship between the TyG-BMI index and the extent of CAD in patients with ACS. The main findings can be summarised as follows: (1) TyG-BMI index is an independent risk factor for mid/high SYNTAX score and linearly correlates with the extent of complex coronary artery disease in female ACS patients; (2) The inclusion of the TyG-BMI index did not improve the predictive power of the baseline risk model in female ACS patients.

IR is an important pathogenetic mechanism in type 2 diabetes [30]. IR usually refers to the reduced sensitivity or responsiveness of the body to insulin metabolism and contributes to the development of CVD through a variety of mechanisms, including alterations in classical CVD risk factors and down-regulation of insulin signalling pathways, with the reduced production of nitric oxide, caused by IR, playing an important role in the development of CVD [5, 31]. The HOMA score is a reliable measure of IR, whereas the TyG index, a simple and easily accessible metric, has recently been recognised as being superior to HOMA in identifying IR [6, 8]. In addition, obesity, defined by BMI, plays an important role in the progression of IR [18]. A study based on a Korean population showed that the TyG-BMI index, calculated using the TyG index and BMI, predicted IR and outperformed the TyG index alone [20], providing new insights into the relationship between the TyG-BMI index and poor cardiovascular prognosis by correcting the TyG index for BMI. Currently, TyG-BMI index is considered a simple, powerful, and clinically useful alternative marker for early detection of IR [27]. However, to date, the relationship between the TyG-BMI index and the extent of CAD in patients with ACS is unclear. In the present study, we focussed on the relationship between the TyG-BMI index and the extent of CAD in patients with ACS and found that the TyG-BMI index was associated with an increased risk of complex CAD among female patients and had some predictive value. It is worth noting that the TyG-BMI index contains three typical CVD risk indicators, i.e., lipid-, glycaemic-, and obesity-related components, and therefore, we performed this analysis to obtain accurate associations. To the best of our knowledge, the current study may be the first to examine the relationship between the TyG-BMI index and the extent of CAD in patients with ACS. In our study, the SYNTAX score was used to quantify coronary disease severity. Further, we adjusted for possible risk factors. Intriguingly, we did not find a significant association between the TyG-BMI index and complex CAD in the entire study population. Therefore, we performed exploratory analyses of sex, age, and DM subgroups. The results showed that in female patients, a higher TyG-BMI index was associated with a greater risk of complex CAD. Previous studies have also shown a correlation between TyG-BMI index and major adverse cardiac and cerebrovascular events (MACCEs) in female patients [32, 33]. Cheng et al. [32] included 2,533 subjects treated with PCI and drug-eluting stent implantation in a retrospective cohort study with the endpoint defined as the composite of acute MI, repeat haemodialysis, stroke, and all-cause mortality at the 34-month follow-up and found that in older and female patients, higher TyG-BMI index was associated with a higher incidence of MACCEs. Similarly, Zhang et al. [33], included 2,533 patients who underwent PCI in a retrospective cohort study with a median follow-up period of 29.8 months, with the endpoint defined as the occurrence of MACCEs and found that the TyG-BMI index was significantly associated with MACCEs in women and in elderly patients. This indirectly validates that TyG-BMI index may be associated with an increased risk of complex CAD in female patients with ACS, which is consistent with the findings of our study. In contrast, there was no significant correlation between TyG-BMI index and complex CAD in male patients. The reasons for this sex difference are unclear but may be partially explained as follows. First, women have a higher burden of cardiovascular risk factors compared to men, and lipid levels and hypertension have a greater adverse effect on women [34]. Second, testosterone is the main male sex hormone, and low testosterone levels are associated with increased cardiovascular risk; hormonal differences between sexes may therefore partly explain the sex differences in patients with ACS and complex CAD [35, 36]. Finally, most women in this study were in the menopausal transition period or in menopause (Additional file 1), and oestrogen has a protective effect on the cardiovascular system [37]. Women are at increased risk of vascular endothelial dysfunction during the menopausal transition or after menopause when the protective effect of oestrogen on the circulatory system is diminished or lost [38, 39]. However, more research is needed to validate this association and investigate its exact mechanism. Therefore, there is an urgent need to conduct further studies to provide more evidence on whether the TyG-BMI index can predict cardiovascular prognosis in patients with ACS. Furthermore, in female patients, the addition of TyG-BMI index to the baseline risk model had no significant incremental effect on predicting the risk of complex CAD. However, unlike a previously cited study, we did not observe an association between TyG-BMI index and an increased risk of complex CAD in elderly patients with ACS, and we hypothesise that this may have been due to the inclusion of younger patients in groups T3 and T2 when compared with that in group T1 as well as an insufficient sample size. Alternatively, this may have been due to the heterogeneity of the included population and the study design. It is worth mentioning that, in this study, the SYNTAX score was used to assess the extent of coronary artery lesions. Based on the current study, the SYNTAX score is an anatomy-based tool that quantifies coronary artery lesions and can be used to make decisions concerning invasive treatment strategies for patients with CAD [40]. An important prognostic and predictive value was shown in patients with acute coronary syndrome [41, 42]. However, SYNTAX scores also have some limitations. First, the SYNTAX score segmentation uses three equal thresholds, and the relationship between these thresholds and clinical outcomes is unclear [43]. Second, SYNTAX scores were not included in the assessment of clinical variables. The presence of clinical comorbidities may affect the long-term and short-term prognosis of patients [44]. Some derivative scores of the SYNTAX score are currently proposed to compensate for its shortcomings. For example, the SYNTAX II score, which combines clinical variables and anatomical SYNTAX scores, was further proposed. The SYNTAX II score predicts the prognosis of CAD patients more accurately and individually [45]. In addition, the Residual SYNTAX score was proposed to help select potential blood supply reconstruction strategies for patients with incomplete haemodialysis [43]. However, the SYNTAX II score and Residual SYNTAX score could not be analysed in depth in this study. This omission prevented the assessment of the correlation between the TyG-BMI index and these scores, limiting the potential prognostic value of the TyG-BMI index for female ACS patients. Future studies should address this gap and investigate the relationship between the TyG-BMI index and SYNTAX-derived scores.

### Strength and limitations

This is the first time that a higher TyG-BMI index is proposed to be linearly associated with the degree of complex coronary artery disease in female ACS patients. However, there are some limitations of this study. First, this was a single-centre study that included only Asian patients, and these results should be interpreted with caution. Second, the current study is limited by its retrospective design, which does not allow causality to be inferred in this study, and further prospective multicentre studies are needed to validate these results. In addition, we cannot completely exclude the possibility of unmeasured or unknown confounders that may explain the associations observed in this study.

## Conclusion

TyG-BMI index is linearly associated with the degree of complex coronary artery disease in female ACS patients. However, the inclusion of the TyG-BMI index did not improve the predictive power of the baseline risk model for female ACS patients. More prospective, large-scale, multicentre studies should be conducted in the future to assess the predictive value of the TyG-BMI index in patients with ACS. In addition, the underlying mechanisms of the linear correlations require further study.

### Electronic supplementary material

Below is the link to the electronic supplementary material.

**Additional file 1.** Baseline characteristics according to sex of the TyG-BMI index

## Data Availability

Due to privacy and ethical constraints, the datasets generated and analysed in this study are not publicly available but can be obtained from the corresponding author.

## Abbreviations

ACS: Acute coronary syndromes
AUC: Area under the curve
BMI: Body mass index
CABG: Coronary artery bypass grafting
CAD: Coronary artery disease
CI: Confidence interval
Cr: Creatinine
CVD: Cardiovascular disease
DBP: Diastolic blood pressure
DM: Diabetes mellitus
eGFR: Estimated glomerular filtration rate
FBG: Fasting blood glucose
Hb: Haemoglobin
HbA1c: Glycosylated haemoglobin
HDL-C: High-density lipoprotein cholesterol
HOMA: Homeostatic model assessment
HOMA-IR: Homeostatic model assessment of IR
hs-CRP: High-sensitivity C-reactive protein
IDI: Integrated Discriminant Improvement Index
IR: Insulin resistance
LDL-C: Low-density lipoprotein cholesterol
LVEF: Left ventricular ejection fraction
MI: Myocardial infarction
NRI: Net Reclassification Index
NSTEMI: Non-ST-segment elevation myocardial infarction
OR: Odds ratio
PCI: Percutaneous coronary intervention
ROC: Receiver operating characteristic
SBP: Systolic blood pressure
STEMI: ST-segment elevation myocardial infarction
TC: Total cholesterol
TG: Triglycerides
TyG: Triglyceride glucose index
