# Supplementary material for: Association of the triglyceride glucose-body mass index with the extent of coronary artery disease in patients with acute coronary syndromes
Source: Cardiovasc Diabetol. 2024 Jan 13;23:24. doi: 10.1186/s12933-024-02124-2 (PMC10790264; doi:10.1186/s12933-024-02124-2)
Supplement: Supplementary file 1 — Additional file 1. Baseline characteristics according to sex of the TyG-BMI index [file 12933_2024_2124_MOESM1_ESM.docx]

**Additional file1. Baseline characteristics according to sex of the TyG-BMI index**

| Sex | Total(N=1,696) | T1(N=565) | T2(N=565) | T3(N=566) | *p* |
| --- | --- | --- | --- | --- | --- |
| Male | 59 (52, 69) | 63(54, 70) | 59 (53, 69) | 55 (50, 66) | <0.001 |
| Female | 68 (59, 74) | 70 (63, 76) | 68 (59, 72) | 66 (57, 71) | 0.002 |
